# PK11195 Protects From Cell Death Only When Applied During Reperfusion: Succinate-Mediated Mechanism of Action

**DOI:** 10.3389/fphys.2021.628508

**Published:** 2021-05-04

**Authors:** Lea K. Seidlmayer, Benjamin J. Hanson, Phung N. Thai, Saul Schaefer, Donald M. Bers, Elena N. Dedkova

**Affiliations:** ^1^Department of Cardiology, University Hospital Olbenburg, Olbenburg, Germany; ^2^Department of Molecular Biosciences, School of Veterinary Medicine, University of California, Davis, Davis, CA, United States; ^3^Department of Internal Medicine, Division of Cardiovascular Medicine, School of Medicine, University of California, Davis, Davis, CA, United States; ^4^Department of Pharmacology, School of Medicine, University of California, Davis, Davis, CA, United States

**Keywords:** TSPO, ischemia-reperfusion, mitochondrial calcium uptake, cell death, PK11195, mitochondrial permeability transition pore, mitochondrial F_1_F_0_-ATPase, Biolog Mitoplates

## Abstract

**Aim**: Reperfusion after myocardial ischemia causes cellular injury, in part due to changes in mitochondrial Ca^2+^ handling, oxidative stress, and myocyte energetics. We have previously shown that the 18-kDa translocator protein of the outer mitochondrial membrane (TSPO) can modulate Ca^2+^ handling. Here, we aim to evaluate the role of the TSPO in ischemia/reperfusion (I/R) injury.

**Methods**: Rabbit ventricular myocytes underwent simulated acute ischemia (20 min) and reperfusion (at 15 min, 1 h, and 3 h) in the absence and presence of 50 μM PK11195, a TSPO inhibitor. Cell death was measured by lactate dehydrogenase (LDH) assay, while changes in mitochondrial Ca^2+^, membrane potential (ΔΨ_m_), and reactive oxygen species (ROS) generation were monitored using confocal microscopy in combination with fluorescent indicators. Substrate utilization was measured with Biolog mitochondrial plates.

**Results**: Cell death was increased by ~200% following I/R compared to control untreated ventricular myocytes. Incubation with 50 μM PK11195 during both ischemia and reperfusion did not reduce cell death but increased mitochondrial Ca^2+^ uptake and ROS generation. However, application of 50 μM PK11195 only at the onset and during reperfusion effectively protected against cell death. The large-scale oscillations in ΔΨ_m_ observed after ~1 h of reperfusion were significantly delayed by 1 μM cyclosporin A and almost completely prevented by 50 μM PK11195 applied during 3 h of reperfusion. After an initial increase, mitochondrial Ca^2+^, measured with Myticam, rapidly declined during 3 h of reperfusion after the initial transient increase. This decline was prevented by application of PK11195 at the onset and during reperfusion. PK11195 prevented a significant increase in succinate utilization following I/R and succinate-induced forward-mode ROS generation. Treatment with PK11195 was also associated with a significant increase in glutamate and a decrease in leucine utilization.

**Conclusion**: PK11195 administered specifically at the moment of reperfusion limited ROS-induced ROS release and cell death, likely in part, by a shift from succinate to glutamate utilization. These data demonstrate a unique mechanism to limit cardiac injury after I/R.

## Introduction

Acute myocardial infarction and ischemic heart disease remain the leading cause of death worldwide (Nowbar et al., 2019). The most effective treatment for acute myocardial infarction is reperfusion therapy, generally through percutaneous coronary intervention or thrombolytic therapy. However, reperfusion therapy itself can cause further injury to myocytes, often through mechanisms such as mitochondrial calcium overload, opening of the mitochondrial permeability transition pore (mPTP), reactive oxygen species (ROS) production, or energetic failure. Pharmacologic interventions at key control points of these mechanisms have failed to provide benefit in clinical trials, raising the need for further understanding of reperfusion injury and development of interventions to reduce it.

We and others have shown that the 18-kDa translocator protein (TSPO), previously known as the peripheral benzodiazepine receptor (PBR), likely plays a key role in cardiac injury during heart failure and myocardial infarction (Mestre et al., 1985; Akar et al., 2005; Brown et al., 2008; Morin et al., 2016; Musman et al., 2017; Thai et al., 2018). TSPO is ubiquitously expressed throughout the body, with elevated expression in steroid-synthesizing tissues, such as adrenal glands and gonads (De Souza et al., 1985; Papadopoulos et al., 1997), as well as the brain (Veenman et al., 2002, 2015) and heart (Veenman and Gavish, 2006; Thai et al., 2018). TSPO is located on the outer mitochondrial membrane and appears to form a multi-protein complex including the voltage-dependent anion channel (VDAC) and the adenine nucleotide transporter (ANT; McEnery et al., 1992; Gatliff et al., 2014). TSPO responds to stress, and its expression is increased in pressure-overload induced heart failure (HF) in mice (Thai et al., 2018) and in circulating monocytes and resident cardiac myocytes in post-myocardial infarction (Thackeray et al., 2018).

Interventions to modulate TSPO expression and function in the heart have had mixed results. We recently demonstrated that genetic cardiospecific ablation of TSPO significantly delayed HF development and improved cardiac bioenergetics in a murine model of pressure overload (Thai et al., 2018). In contrast, pharmacologic interventions with TSPO agonists and antagonists have yielded conflicting results in models of I/R, showing either a protective effect of TSPO inhibitors (Mestre et al., 1985) or suppression of cardiac function (Shany et al., 1994).

Given these mixed data, we examined effect of PK11195, an isoquinoline carboxamide, which is known to bind (Jaremko et al., 2014) and inhibit TSPO (Le Fur et al., 1983a,b; Mestre et al., 1985), on its ability to limit ischemia/reperfusion (I/R) injury (IRI) in a cellular model of simulated I/R. Exposure of freshly isolated cardiomyocytes to PK11195 either at the onset of I/R or only during reperfusion, with assessment of cell death, ROS production, and energetics, provided insight into the mechanism by which PK11195 may be beneficial. In brief, PK11195 was protective only when applied during reperfusion, likely by decreasing the use of succinate with resultant better maintenance of mitochondrial Ca^2+^ handling and lower ROS production. Furthermore, PK11195 strongly inhibited ROS-induced ROS release (RIRR) and cell death when applied at reperfusion. This effect of PK11195 was comparable and even stronger compared to the known mPTP desensitizer, cyclosporin A (CsA), suggesting inhibition of mPTP by PK11195. Given the fact that some studies reported the inhibitory effect of PK11195 on the c subunit of the mitochondrial ATP synthase complex (Cleary et al., 2007; Krestinina et al., 2009), it is plausible that PK11195 may exert TSPO-independent protective effects *via* inhibition of the c subunit ATP synthase, which is currently considered as a pore component of the mPTP. Further studies will be required to evaluate this possibility.

## Materials and Methods

### Animal Model

All animal handling and laboratory procedures were performed in accordance with the approved protocols of the Institutional Animal Care and Use Committee of the University of California, Davis conforming to the Guide for the Care and Use of Laboratory Animals published by the United States National Institute of Health (8th Edition, 2011).

### Cell Isolation

For cardiomyocyte isolation, rabbits were subjected to general anesthesia induced with 2 mg/kg propofol followed by 2–5% isoflurane in 100% oxygen (Dedkova et al., 2013; Thai et al., 2018). After anesthesia was verified, thoracotomy was conducted, and the heart was quickly removed and rinsed in cold nominally Ca^2+^-free minimal essential medium (MEM). The right atrium was excised and the aorta opened to visualize the left coronary ostium, which was then cannulated using a 5-F Judkins right catheter (Performa; Merit Medical Systems). Perfusion of the left ventricle and left atrium was established before removal of the right ventricular free wall and application of a purse-string suture to secure the catheter in place. Then, the heart was perfused with MEM solution containing 20 μmol/l Ca^2+^ and 22.5 μg/mg Liberase TH at 37°C until tissue was digested. The left ventricle was cut from the heart and minced, filtered, and washed in a MEM solution containing 50 μmol/l Ca^2+^ and 10 mg/ml bovine serum albumin. Isolated cells were kept in MEM solution with 50 μmol/l Ca^2+^ at room temperature (22–24°C) until used for experimentation or placed in a short-term cell culture (see below).

### Simulated Ischemia/Reperfusion Protocol

Ischemia was simulated by acidosis (pH 6.4), inhibition of glycolysis (glucose replaced with 20 mM deoxyglucose), and inhibition of mitochondrial respiration (with complex IV inhibitor sodium cyanide, NaCN) using glucose-free modified Tyrode solution containing (in mM) 20 2-deoxyglucose, 2 NaCN, 135 NaCl, 4 KCl, 1 MgCl_2_, 2 CaCl_2_, and 10 Hepes, pH 6.4 for 20 min (Seidlmayer et al., 2015). Reperfusion was simulated by 15 min, 1 h, and 3 h superfusion with standard Tyrode solution consisting of (in mM) 135 NaCl, 4 KCl, 10 glucose, 10 Hepes, 1 MgCl_2_, and 2 CaCl_2_; pH 7.4.

### [Ca^2+^]_m_ Measurements

Changes in mitochondrial Ca^2+^ concentration ([Ca^2+^]_m_) were measured in freshly isolated ventricular myocytes using the Ca^2+^-sensitive fluorescent probe X-Rhod-1/AM (λ_ex_ = 543 nm; λ_em_ = 552–617 nm; Seidlmayer et al., 2015) or in cultured primary ventricular myocytes with expressed Ca^2+^ sensitive protein Mitycam (λ_ex_ = 488 nm; λ_em_ = 530 nm; Kettlewell et al., 2009; Dedkova and Blatter, 2012). Fluorescence was monitored using a Nikon A1 laser scanning confocal microscope. For Mitycam expression, freshly isolated ventricular myocytes were plated on laminin-coated sterile glass cover slips for 1 h before exposure to the adenoviral-construct Mitycam at a multiplicity of infection (MOI) of 500 virus particles per cell (vp/cell). Infected cells were cultured in serum-free PC-1 medium for 24 h. To prevent detubulation and functional changes in cardiomyocytes during short-term culture, cells were electrically stimulated at 0.2 Hz during the culture.

For [Ca^2+^]_m_ measurements with X-Rhod-1, cardiomyocytes were loaded with X-Rhod-1/AM for 30 min at 37°C. This loading procedure favors mitochondrial localization of X-Rhod-1. To quench the remaining cytosolic fluorescence, cells were incubated in 1 mM CoCl_2_-containing Tyrode during washout period as published before (Seidlmayer et al., 2015, 2016).

### Mitochondrial Membrane Potential Measurements

Mitochondrial membrane potential (ΔΨ_m_) was monitored in intact cardiomyocytes using the potentiometric probe tetramethylrhodamine methyl ester (TMRM; λ_ex_ = 543 nm and λ_em_ = 565–605 nm; Dedkova and Blatter, 2012; Seidlmayer et al., 2015, 2016). Cardiomyocytes were loaded with 5 nM TMRM for 30 min at 37°C. About 5 nM TMRM was present in all solutions during the experiments. Changes in TMRM fluorescence were recorded every 1 min to minimize photobleaching of samples. At the end of each experiment, 10 μM FCCP and 1 μM oligomycin were added to calibrate the signal. Background was subtracted from all data. Data were normalized to pre-ischemia levels.

Cell death measurements were performed by counting cells which shrunk at the end of experiments and had nuclei positively stained with propidium iodide (PI; λ_ex_ = 555 nm and λ_em_ = 605 nm). PI is a cell impermeable fluorescent dye which binds to double-stranded DNA. Therefore, PI will only stain DNA in cells where the plasma membrane has been damaged. About 500 nM propidium iodide was added in the end of reperfusion.

### ROS Generation

Reactive oxygen species generation was measured in freshly isolated myocytes loaded with 0.5 μM MitoSox Red (λ_ex_ = 543 nm, λ_em_ = 555–617 nm) for 30 min at 37°C (Seidlmayer et al., 2015). Changes in MitoSox Red fluorescence intensity (F) during I/R were normalized to the level of fluorescence recorded before ischemia (F_0_), and expressed as ΔF/F_0_ or as rate of fluorescence increase normalized to basal level. Changes in MitoSox Red fluorescence were recorded every 1 min.

### Mitochondrial Substrate Utilization

Mitochondrial substrate utilization was assayed by measuring the rates of electron flow into and through the electron transport chain from metabolic substrates that produce NAD(P)H or FADH_2_ using Biolog Mitochondrial Plates (Hayward, CA, United States). For this assay, freshly isolated cardiomyocytes were counted, resuspended at 1 × 10^6^ cells/ml density and split into three groups. One group was kept in Tyrode solution, and the two other groups were exposed to simulated ischemia for 20 min. After 20 min, control or ischemia solutions were removed, and cells from the three groups were resuspended in Biolog mitochondrial assay solution (MAS) containing 30 μg/ml saponin and the Redox Dye MC (Biolog, 74353) in the absence or presence of 50 μM PK11195. Next, 30 μl of cells was added into the wells preloaded with different cytosolic and mitochondrial metabolites. Each substrate follows a different route, using different transporters to enter the mitochondria and different dehydrogenases to produce NAD(P)H or FADH_2_. The electrons travel from the beginning (complex I or II) to the distal portion of the electron transport chain where a tetrazolium redox dye acts as a terminal electron acceptor that turns purple upon reduction. Absorbance at 590 nm was measured using BioTek Synergy Mx multiplate reader. The initial rate of absorbance increase was measured in each experimental group.

### Cell Death Measurement

Cell death was measured by the lactate dehydrogenase (LDH) release into the extracellular medium after 20 min of simulated ischemia and 15 min, 1 h, and 3 h of reperfusion (Seidlmayer et al., 2015). The LDH assay based on the conversion of yellow tetrazolium salt by LDH into a red, formazan-class dye was used according to manufacturer specifications (Clontech). Absorbance was measured spectrophotometrically using BioTek Synergy Mx multiplate reader at 490 nm. Non ischemia-reperfusion time dependent controls were performed for each experimental condition (i.e., in the absence and presence of PK11195). Percent of LDH release was calculated as an increase in LDH absorbance normalized to the time-dependent controls. This normalization allows accounting for cell death induced by mechanical stress due to solution replacement and suction.

### Statistical Analysis

All data were analyzed using unpaired *t*-tests or two-way analysis of variance with Tukey’s *post-hoc*, using GraphPad Prism version 8.0 (Graph-Pad Software Inc., San Diego, United States) and *p* < 0.05 was considered statistically significant. All data are reported as mean +/−SEM.

## Results

### PK11195 Protected Myocytes From I/R-Induced Cell Death Only When Applied During Reperfusion

**Figure 1:**
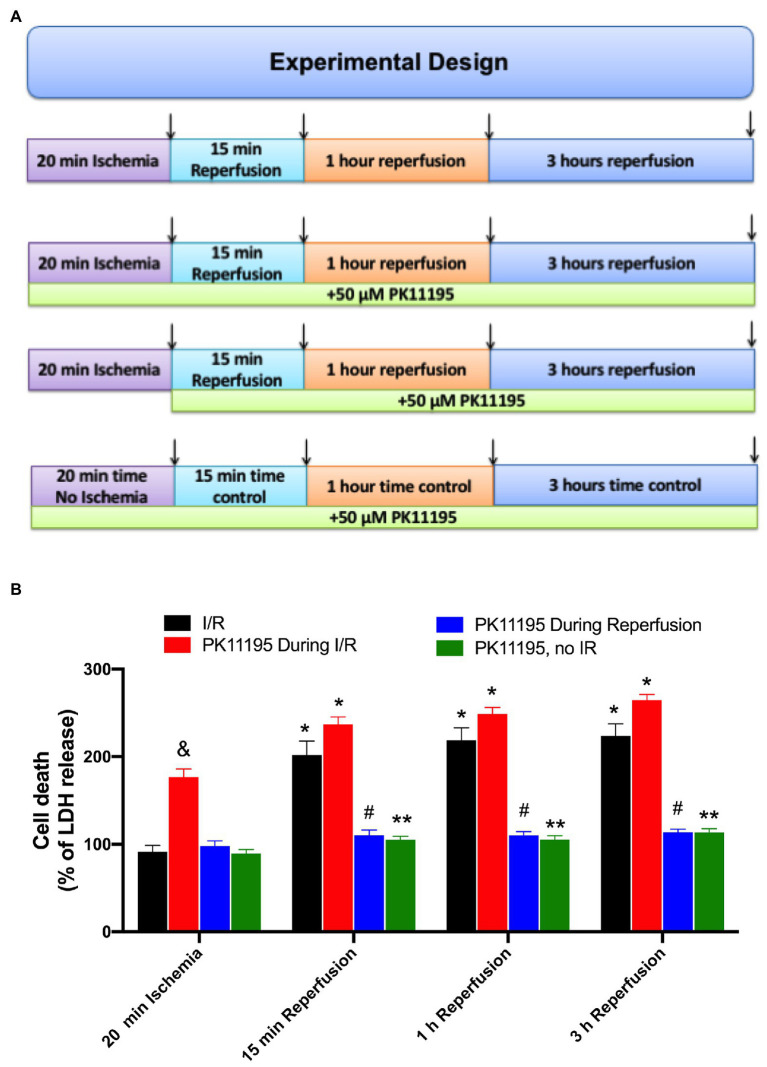
Effects of PK11195 compound on cell death in conditions of simulated ischemia and reperfusion. **(A)** Schematic representation of experimental design. Freshly isolated ventricular myocytes were exposed to 20 min of simulated ischemia, followed by simulated reperfusion as shown in the upper panel. At the end of 20 min ischemia, and 15 min, 1 h, and 3 h of reperfusion, samples were collected for lactate dehydrogenase (LDH) detection as shown by arrows. Control experiments were done in the absence of 50 μM PK11195, while in the second set of experiments, 50 μM PK11195 was present during both simulated ischemia and reperfusion, and in the third set of experiments, 50 μM PK11195 was added only during reperfusion phase. The fourth set of experiments was a time no I/R control in the presence of 50 μM PK11195. **(B)** Cell death following ischemia-reperfusion as measured by LDH release was significantly decreased by 50 μM PK11195 only when added at the onset of the reperfusion. Green bars represent time control in the presence of 50 μM PK11195 but in the absence of I/R. Data expressed as mean ± SEM. *n* = number of cell preparation from six different animals per experimental group. ^*^p < 0.05 reflects the increase in cell death in reperfusion vs. ischemia in each group; ^#^*p* < 0.001 reflects the decrease in cell death in PK11195-treated cells during reperfusion vs. untreated cells; ^&^*p* < 0.001 reflects the increase in cell death in PK11195 treated cells during ischemia vs. ischemia in the absence of PK11195 in each group; ^**^p < 0.001 reflects the difference between PK11195, no I/R vs. I/R and PK11195 during I/R.

To evaluate the effect of PK11195 on cellular death during I/R, freshly isolated rabbit ventricular myocytes were exposed to 20 min of simulated ischemia followed by 15 min, 1 h, and 3 h reperfusion time as shown in Figure 1A. At the end of ischemia and each reperfusion period, samples were collected, and the amount of LDH released to the medium was measured as previously described (Chan et al., 2013; Seidlmayer et al., 2015). All data were normalized to the corresponding time controls (no I/R) and are represented as percent of LDH release relative to no I/R time controls. As shown in Figure 1B, no LDH release was detected following 20 min of ischemia; however, the amount of LDH release (i.e., cell death) was increased almost 2-fold after 15 min of reperfusion (202 ± 16 vs. 92 ± 7% during ischemia, *n* = 12) and remained at the same level after 1 h (219 ± 14%) and 3 h (224 ± 14%) of reperfusion (black bars). Cell treatment with 50 μM PK11195 during both ischemia and reperfusion did not prevent cell death on reperfusion, and even exacerbated cell death during ischemia itself (178 ± 10%, *n* = 10 vs. 92 ± 7% during ischemia not exposed to PK11195, *n* = 12, *p* < 0.001, red bars). However, 50 μM PK11195 exposure only at the onset of reperfusion effectively prevented cell death at 15 min (103 ± 11%, *n* = 10, *p* < 0.001), 1 h (110 ± 6%, *n* = 10, *p* < 0.001), and 3 h (121 ± 14%, *n* = 10, *p* < 0.001) reperfusion time (blue bars). Time control performed in the absence of ischemia demonstrated that 50 μM PK11195 by itself did not induce cell death (green bars). Therefore, these data suggest that 50 μM PK11195 was amplifying some signaling pathways leading to cell death during ischemia, possibly Ca^2+^ or ROS or both.

### PK11195 Applied at the Onset of Reperfusion Prevented a Drop in Mitochondrial Ca^2+^ During Prolonged Reperfusion

**Figure 2:**
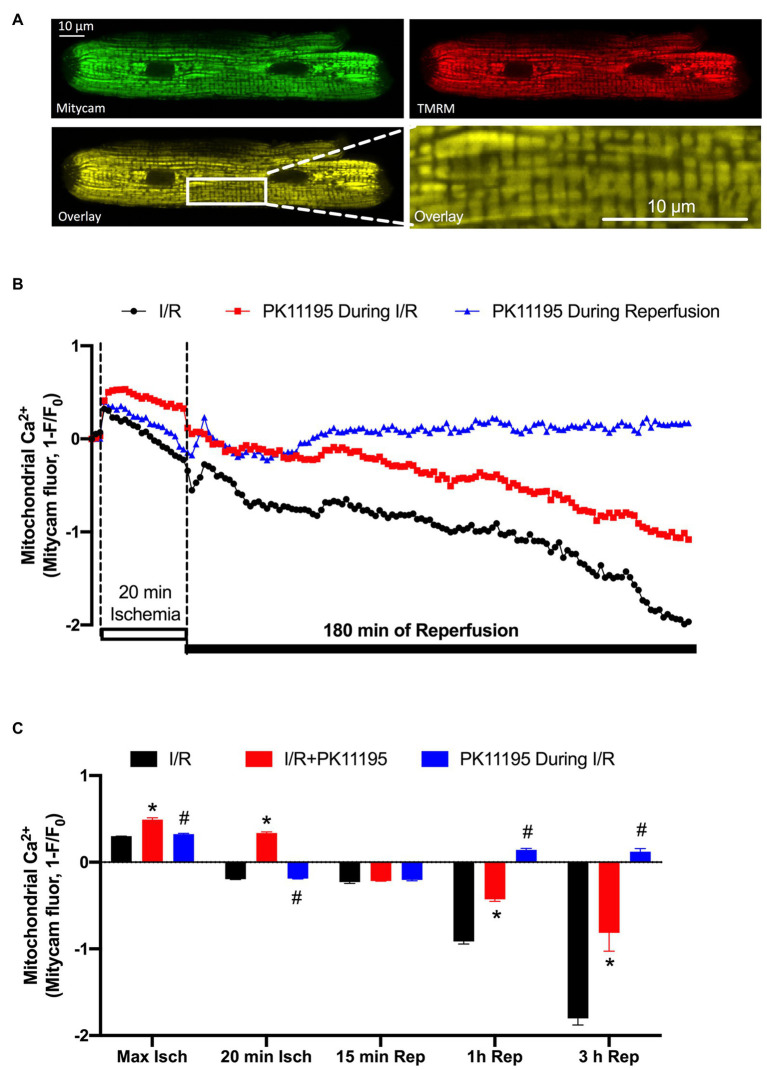
Changes in mitochondrial Ca^2+^ concentration ([Ca^2+^]_m_) during 20 min of simulated ischemia followed by 3 h of reperfusion. **(A)** Shown are the images of cardiomyocyte with expressed Ca^2+^-sensitive probe Mitycam (green) and loaded with 50 nM TMRM (red), and an overlay of both channels (yellow) confirming mitochondrial location of Ca^2+^ protein sensor Mitycam. Expanded image on the right indicates enlarged mitochondrial region from the left image. **(B)** Changes in mitochondrial Ca^2+^ during I/R monitored by Mitycam in the absence (black trace) or presence of 50 μM PK11195 applied either during both ischemia and reperfusion (red trace) or only during reperfusion (blue trace). **(C)** Summary of mitochondrial Ca^2+^ during I/R in the absence or presence of 50 μM PK11195 applied during both I/R or only during reperfusion. Measurements were performed at the maximal [Ca^2+^]_m_ elevation in the beginning of ischemia (Max Isch), and in the end of 20 min ischemia, and 15 min, 1 h, and 3 h of reperfusion. Data expressed as mean ± SEM. *n* = number of cell preparations from 3 to 4 different animals per experimental group. ^*^p < 0.05 reflects the difference between PK11195-treated cells vs. untreated cells exposed to I/R; ^#^*p* < 0.05 reflects the difference between PK11195-treated cells applied during reperfusion vs. untreated cells exposed to I/R.

We have previously demonstrated that cardio-specific TSPO KO restored diminished mitochondrial Ca^2+^ uptake under conditions of heart failure (Thai et al., 2018). We, therefore, tested whether inhibition of TSPO with 50 μM PK11195 could maintain mitochondrial Ca^2+^ uptake during I/R. To monitor [Ca^2+^]_m_ changes during our long protocol for ischemia-reperfusion (more than 3 h), the mitochondrially targeted Ca^2+^-sensitive fluorescent protein, Mitycam (Kettlewell et al., 2009), was adenovirally expressed in ventricular myocytes. The proper mitochondrial targeting of Mitycam was confirmed by colocalization with mitochondria-entrapped TMRM (Figure 2A). Mitycam fluorescence decreases upon binding to Ca^2+^, and we, therefore, present our data as an inverted (1−F/F_0_), such that increases in [Ca^2+^]_m_ are an upward deflection (Kettlewell et al., 2009). Figure 2B shows a transient increase in mitochondrial [Ca^2+^]_m_ shortly after the onset of ischemia (from 0 baseline to 0.301 ± 0.001, *n* = 10). [Ca^2+^]_m_ then progressively declined to levels below baseline, reaching normalized levels of −0.196 ± 0.003, *n* = 10. The initial decline might reflect a recovery of mitochondrial Ca^2+^ overload, but the further fall below baseline might also reflect some loss of mitochondrial integrity (i.e., mPTP opening). Upon reperfusion, [Ca^2+^]_m_ rose transiently, as expected, but then continuously declined during 3 h of reperfusion (Figure 2C).

Treatment of cardiomyocytes with 50 μM PK11195 during both ischemia and reperfusion, significantly increased mitochondrial [Ca^2+^]_m_ during ischemia (0.492 ± 0.02, *n* = 10, *p* < 0.001), but still resulted in major progressive [Ca^2+^]_m_ decline during the reperfusion (−0.814 ± 0.212, *n* = 10) paralleling that seen without drug. However, when 50 μM PK11195 was introduced only during the reperfusion phase, it prevented the progressive [Ca^2+^]_m_ decline and stabilized [Ca^2+^]_m_ slightly above the baseline value (0.121 ± 0.037, *n* = 10, *p* < 0.001). These data suggest that PK11195 administration during the reperfusion phase protected against the rapid [Ca^2+^]_m_ decline, possibly by limiting mPTP opening or promoting MCU-dependent mitochondrial Ca^2+^ uptake with the subsequent improvement in mitochondrial energetics.

### PK11195 Prevented Mitochondrial Membrane Potential Oscillations During 3 Hours of Reperfusion

**Figure 3:**
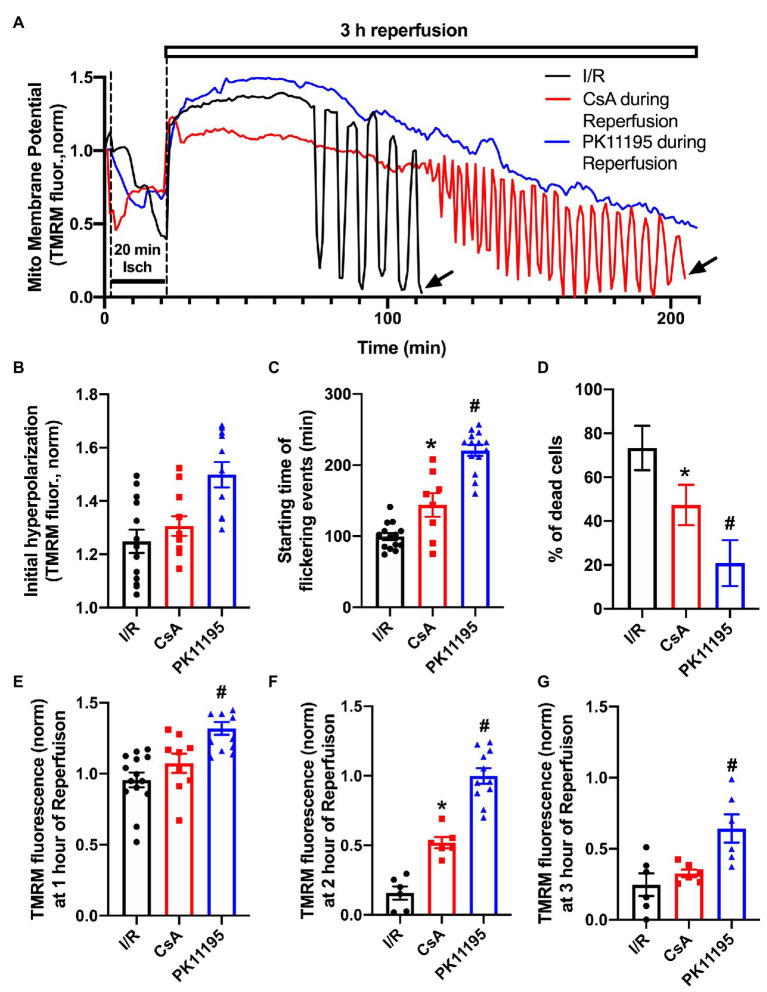
Changes in mitochondrial membrane potential during simulated ischemia and reperfusion. **(A)** Shown are the traces reflecting changes in mitochondrial membrane potential during ischemia and reperfusion in the absence or presence of 1 μM cyclosporin A (CsA) and 50 μM PK11195 added at the onset of reperfusion. The abrupt ending in black and red tetramethylrhodamine methyl ester (TMRM) traces was due to cell death as indicated by the black arrows. **(B)** Summary data of the initial hyperpolarization at the onset of reperfusion. **(C)** Starting time of flickering events. **(D)** The percentage of dead cells observed during reperfusion compared to the pre-ischemia/reperfusion (I/R) levels. **(E-G)** The normalized TMRM levels measured at 1, 2, and 3 h of reperfusion. Data expressed as mean ± SEM. *n* = 3–5 animals per group. ^*^p < 0.05 reflects a difference between untreated I/R cardiomyocytes and cardiomyocytes treated with 1 μM CsA at the onset of reperfusion; ^#^*p* < 0.05 reflects a difference between untreated I/R cardiomyocytes and cardiomyocytes treated with 50 μM PK11195 at the onset of reperfusion.

It is known that restoration and maintenance of the mitochondrial inner membrane potential (ΔΨ_m_) following an ischemic event plays a major role in postischemic functional recovery of the heart (Akar et al., 2005; Aon et al., 2006). We, therefore, monitored changes in ΔΨ_m_ using the membrane-potential sensitive dye TMRM. Cardiomyocytes attached to the glass coverslips were loaded with 5 nM TMRM for 30 min at 37°C, and then coverslips were transferred to the stage of the confocal microscope and baseline ΔΨ_m_ was recorded. Since TMRM is a re-distribution positively charged fluorescent dye which accumulates in negatively charged membranes according to the Nernstian equation, TMRM was present in all experimental solutions to maintain its concentration during cell perfusion. During the 20 min ischemic period ΔΨ_m_ dropped by ~50% compared to pre-ischemic values (Figure 3A). Upon the onset of reperfusion, ΔΨ_m_ recovered quickly and reached the levels well above the pre-ischemic values (Figure 3B). However, over the time, ΔΨ_m_ started to flicker with a large-scale ΔΨ_m_ oscillations ~40–100 min post reperfusion time. Figure 3C shows that these oscillations were significantly delayed by the mPTP inhibitor, cyclosporin A, and almost completely abolished by PK11195 treatment applied during reperfusion. The vast majority of control cells exposed to I/R (i.e., untreated with drugs) died prematurely before the end of 3 h of reperfusion. As shown in Figure 3A, TMRM fluorescence recording ended abruptly in I/R representative trace (black) due to myocyte death around 110 min as indicated by the arrow. A similar premature death was also observed in CsA-treated cells (red trace) following large-scale oscillations but at a much later time (~210 min, black arrow). On average, the percent of dead cells counted at the end of 3 h reperfusion decreased from 70% in I/R group to less than 20% in PK11195 treated group (*p* = 0.004; Figure 3D). Cell death measurements were performed by counting cells which had shrunken (balled-up by hypercontracture) at the end of experiments and which also had nuclei positively stained with propidium iodide (PI) which is excluded from intact cells, but binds to double-stranded DNA. Therefore, PI will stain DNA only in cells with a compromised plasma membrane. The protective effect of CsA treatment was significantly smaller compared to PK11195-treated group (Figure 3D). This was also evident by the mean TMRM fluorescence values (i.e., ΔΨ_m_) measured at 1, 2, and 3 h of reperfusion time (Figures 3E–G). At 1-h post-reperfusion time, mean TMRM fluorescence values were still maintained at the levels close to pre-ischemic ΔΨ_m_ in all three groups (Figure 3E). While CsA protected cells against ΔΨ_m_ drop at 2 h of reperfusion time (Figure 3F), it failed to protect against a drop in ΔΨ_m_ at 3 h of reperfusion (Figure 3G). At the same time, ΔΨ_m_ was maintained at a higher level with 50 μM PK11195 treatment at 1, 2, and 3 h of monitored reperfusion time (Figures 3E–G). These data demonstrate that TSPO inhibitor PK11195 effectively prevented destabilization in ΔΨ_m_ when applied during reperfusion.

### PK11195 Inhibited Reactive Oxygen Species Production During I/R

**Figure 4:**
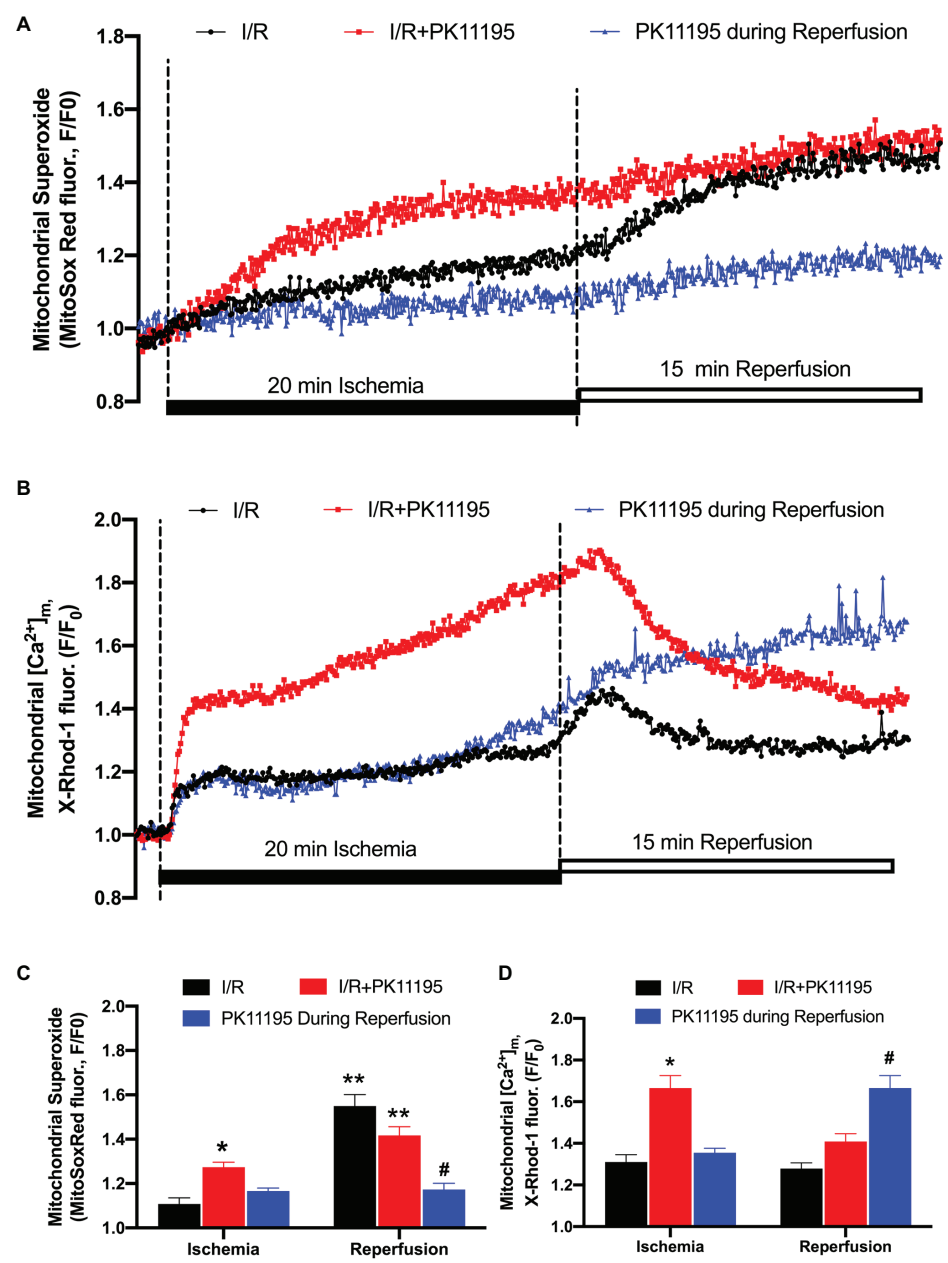
Changes in reactive oxygen species (ROS) generation and mitochondrial Ca^2+^ concentration during simulated ischemia and reperfusion. **(A)** Representative traces of superoxide generation measured with MitoSox Red during I/R in the absence of 50 μM PK11195 (black) and when 50 μM PK11195 was added during the whole period of I/R (red) or added during a reperfusion phase (blue). **(B)** Changes in mitochondrial Ca^2+^ measured with X-Rhod-1 during I/R in the absence of 50 μM PK11195 (black) and when 50 μM PK11195 was added during the whole period of I/R (red) or added during a reperfusion phase (blue). **(C,D)** Effects of PK11195 on mitochondrial ROS and [Ca^2+^]_m_ during I/R when added during I/R (red) or at the onset of reperfusion (blue). Data expressed as mean ± SEM. *n* = 3–5 animals per group. ^*^p < 0.05 reflects a difference between untreated I/R cardiomyocytes and cardiomyocytes which were treated with 50 μM PK11195 during I/R; ^#^*p* < 0.05 reflects a difference between untreated I/R cardiomyocytes and cardiomyocytes which were treated with 50 μM PK11195 at the onset of reperfusion; ^**^p < 0.05 reflects the difference between the corresponding ischemia and reperfusion sets.

It has been previously suggested that mitochondrial RIRR leads to the collapse of ∆Ψ_m_ (Zorov et al., 2000) and the destabilization of the action potential (AP; Akar et al., 2005). We, therefore, measured levels of ROS generated during simulated ischemia and reperfusion using the fluorescent dye MitoSox Red. As shown in Figure 4A, ROS levels were already elevated during 20 min of ischemia, and further increased during the reperfusion phase. Incubation with 50 μM PK11195 throughout I/R substantially increased ROS generation during ischemia and reperfusion. However, when PK11195 was added only at the onset of reperfusion, ROS generation was significantly decreased (Figure 4C).

A potential explanation for the surprising increase in ROS when PK11195 was given during ischemia could be an enhanced mitochondrial Ca^2+^ accumulation as a consequence of TSPO inhibition. This would be consistent with data in Figure 2 where [Ca^2+^]_m_ remained elevated during ischemia in the presence of PK11195. To further test this possibility, we monitored [Ca^2+^]_m_ with the fluorescent indicator X-Rhod-1 which has a higher K_d_ = 700 nM for Ca^2+^. This also overcomes the limitation that Mitycam advenoviral expression requires culturing cardiomyocytes for 24–48 h, which might alter myocyte function. Using X-Rhod-1 allowed us to monitor changes in [Ca^2+^]_m_ in the same experimental conditions (i.e., freshly isolated ventricular myocytes) as used for measurement of ROS shown in Figure 4A. Similar to the data obtained with Mitycam, [Ca^2+^]_m_ increased during ischemia (Figure 4B). As in the control myocytes, a transient increase in [Ca^2+^]_m_ was observed on reperfusion, followed by a decline in [Ca^2+^]_m_. When 50 μM PK11195 was present during both ischemia and reperfusion, an almost 2-fold increase in [Ca^2+^]_m_ during ischemia was observed. However, there was an increase in [Ca^2+^]_m_ at the onset of the reperfusion, followed by a rapid decline in [Ca^2+^]_m_ (Figure 4B). In contrast, when 50 μM PK11195 was added at the onset of the reperfusion, [Ca^2+^]_m_ remained above that observed during ischemia and did not decline (Figure 4D) consistent with the lack of increase in mitochondrial ROS during reperfusion (Figure 4C). These data demonstrate that a TSPO inhibitor helps to maintain pre-ischemic levels of [Ca^2+^]_m_ and prevents ROS generation only when added at the onset of reperfusion.

### Increased Succinate Utilization Following I/R Was Decreased by PK11195

Previous studies have suggested that an increased accumulation of succinate during I/R drives reperfusion injury (Chouchani et al., 2014; Zhang et al., 2018). Here, we examined whether PK11195 administration at the onset of reperfusion affected mitochondrial substrate utilization following simulated I/R. For this purpose, we used Biolog MitoPlates preloaded with different cytosolic and mitochondrial substrates. This assay measured the rates of electron flow through the electron transport chain from metabolic substrates that produce NAD(P)H or FADH_2_ (complex I and complex II) to cytochrome c where a tetrazolium redox dye acts as a terminal electron acceptor that turns purple upon reduction.

**Figure 5:**
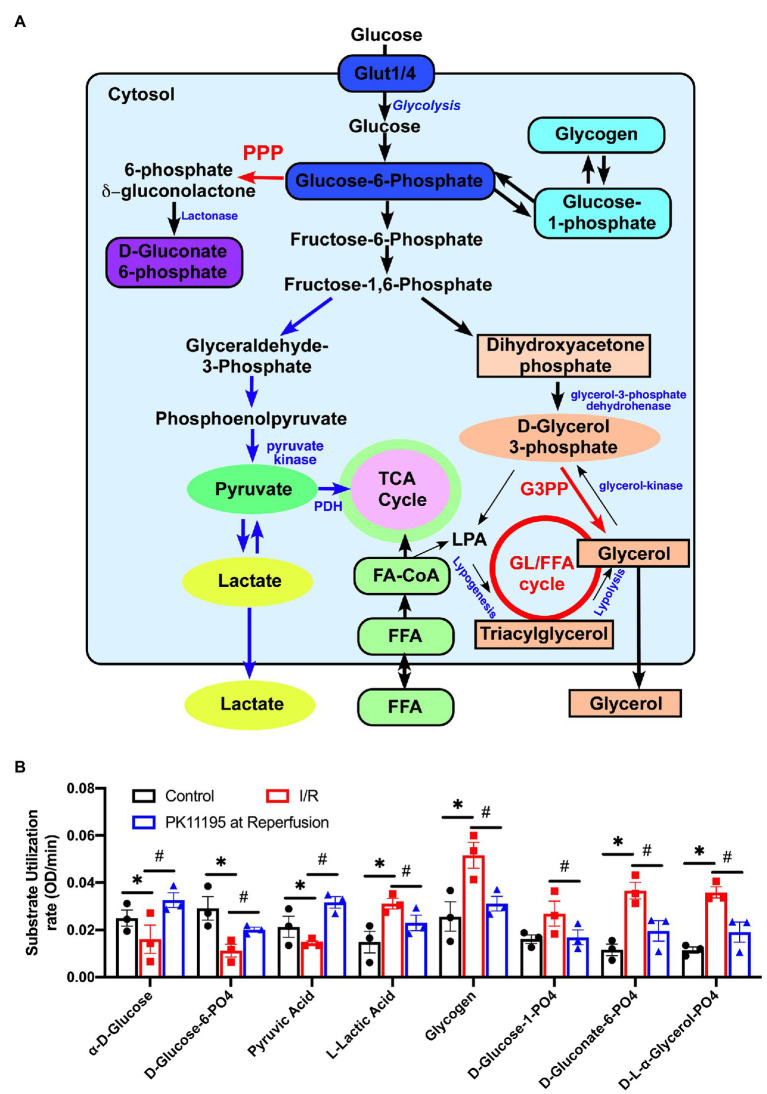
Changes in utilization of glycolytic substrates and intermediates in untreated rabbit cardiac myocytes, and cardiomyocytes exposed to simulated I/R in the absence or presence of 50 μM PK11195. **(A)** Schematic representation of the intermediates of glycolysis, pentose phosphate pathway, glycogenolysis, and the gluconeogenesis pathway. GL/FFA cycle, glycerolipid/free fatty acid cycle; LPA, lysophosphatidic acid; 3GPP, glycerol-3-phosphate phosphatase; PPP, pentose phosphate pathway, PDH, pyruvate dehydrogenase. **(B)** Shown are changes in utilization of the intermediates involved in glycolysis, pentose phosphate, glycogenolysis, and the gluconeogenesis pathways measured with Biolog MitoPlates. Control rabbit cardiomyocytes were exposed to 20 min of simulated ischemia and then exposed to simulated reperfusion in the absence or presence of 50 μM PK11195. Data presented as mean ± SEM obtained from three different animals per each group. ^*^p < 0.05 reflects a significant difference in intermediate utilization between control cardiomyocytes and cardiomyocytes exposed to simulated I/R; ^#^*p* < 0.05 reflects a significance between PK11195 treated cardiomyocytes subjected to simulated I/R vs. cardiomyocytes exposed to I/R in the absence of PK11195. There was no difference between control and PK11195 at reperfusion groups.

**Figure 6:**
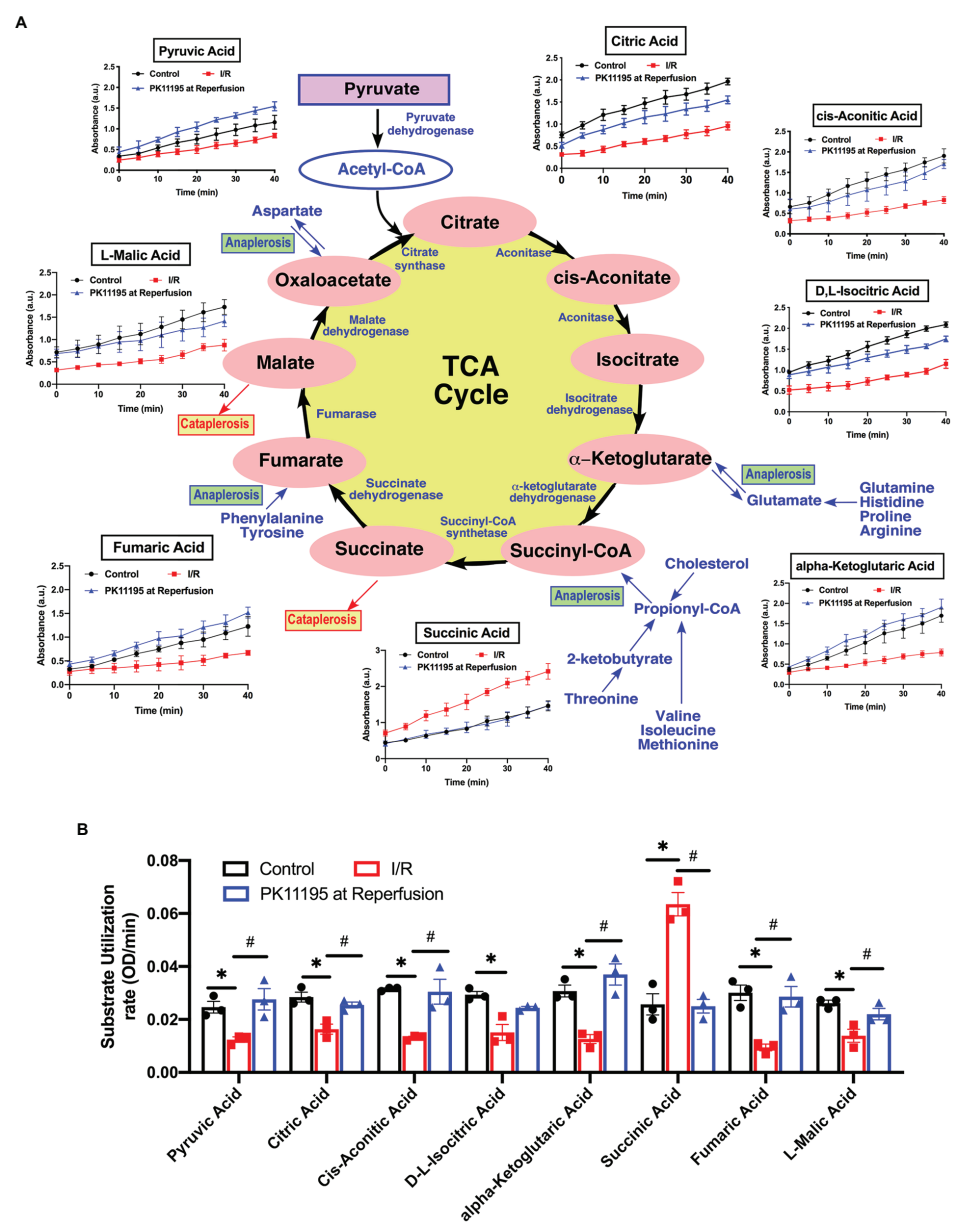
Changes in Krebs (TCA) cycle substrate utilization in untreated rabbit cardiac myocytes, and cardiomyocytes exposed to simulated I/R in the absence or presence of 50 μM PK11195. **(A)** Schematic representation of the intermediates in TCA cycle with the changes in intermediates utilization measured in the Biolog Mito plates. Traces show changes in absorbance measured at 490 nm upon cells addition to the Biolog plates pre-loaded with substrate. All substrates were available at time 0 of the “simulated” reperfusion and present during 40 min of recording. About 50 μM PK11195 was added during simulated reperfusion. **(B)** Summary bars reflected changes of TCA intermediates utilization rates in control rabbit cardiomyocytes and cardiomyocytes exposed to 20 min of simulated ischemia and then exposed to simulated reperfusion in the absence or presence of 50 μM PK11195. Data presented as mean ± SEM obtained from three different animals per each group. ^*^p < 0.05 reflects a significant difference in intermediate utilization between control cardiomyocytes and cardiomyocytes exposed to simulated I/R; ^#^*p* < 0.05 reflects a significance between PK11195 treated cardiomyocytes subjected to simulated I/R vs. cardiomyocytes exposed to I/R in the absence of PK11195. There was no difference between control and PK11195 at reperfusion groups.

**Figure 7:**
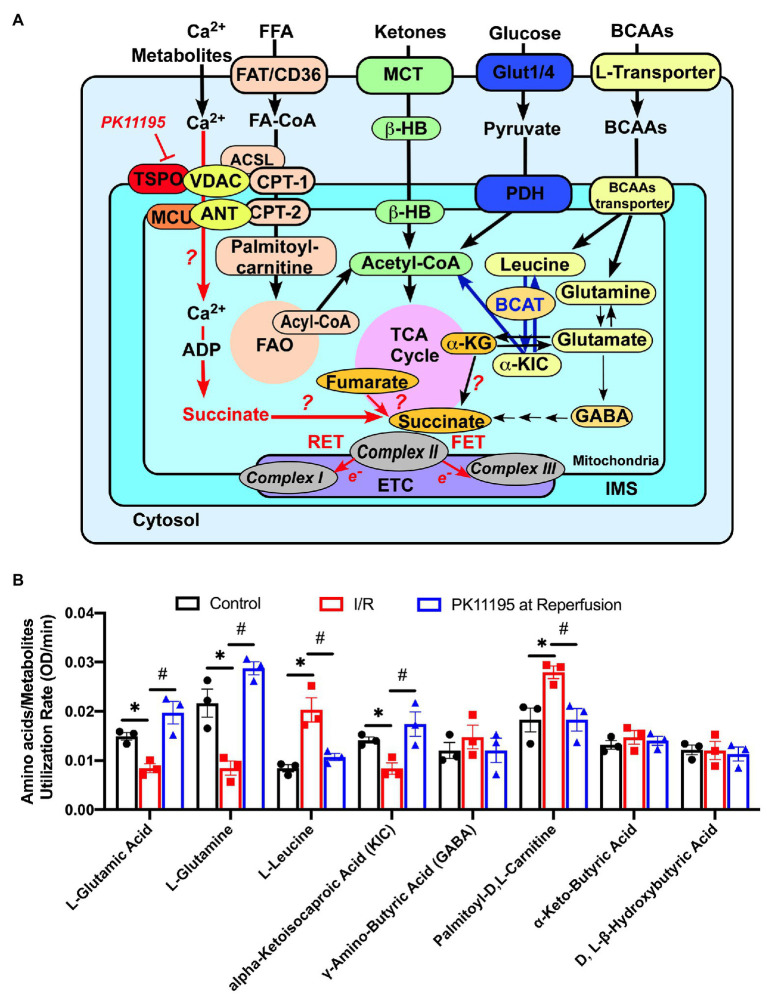
Changes in amino acids, lipid metabolites, and ketone utilization in untreated rabbit cardiac myocytes, and cardiomyocytes exposed to simulated I/R in the absence or presence of 50 μM PK11195. **(A)** Schematic representation of amino acids, ketones, glucose, and lipid metabolites utilization in cardiac myocytes. The increase in succinate during I/R could be due to (1) increased supply of glutamate *via* α-ketoglutarate by transamination, (2) partial reversal of mitochondrial TCA cycle during ischemia and complex II working as fumarate reductase; or (3) potentially by increased delivery of succinate to mitochondria *via* VDAC. Inhibition of translocator protein of the outer mitochondrial membrane (TSPO) with PK11195 could decrease succinate transport *via* VDAC and increase mitochondrial Ca^2+^ uptake at the same time. For simplicity purpose, mitochondrial complexes IV and V of the electron transport chain (ETC) are not shown. ACSL, long chain acyl-CoA synthetase; BCAT, branched-chain aminotransferase enzyme; β-HB, D, L, β-Hydroxybutyric acid; CPT-1, mitochondrial carnitine palmitoyltransferase 1; CPT-2, mitochondrial carnitine palmitoyltransferase 2; FAO, fatty acid oxidation; FAT/CD36, fatty acid transporters; Glut1/4, glucose transporter; α-KG, α-ketoglutarate; α-KIC, α-ketoisocaproic acid; MCT, monocarboxylate transporter; MCU, mitochondrial Ca^2+^ uniporter, VDAC, voltage-dependent anion channel. **(B)** Summary bars reflected changes in amino acids and metabolites utilization rates in control rabbit cardiomyocytes and cardiomyocytes exposed to 20 min of simulated ischemia and then exposed to simulated reperfusion in the absence or presence of 50 μM PK11195. Data presented as mean ± SEM obtained from three different animals per each group. ^*^p < 0.05 reflects a significant difference between control cardiomyocytes and cardiomyocytes exposed to simulated I/R; ^#^*p* < 0.05 reflects a significance between PK11195 treated cardiomyocytes subjected to simulated I/R vs. cardiomyocytes exposed to I/R in the absence of PK11195. There was no difference between control and PK11195 at reperfusion groups.

Before loading into the plates, cells were exposed to simulated ischemia or control Tyrode solution for 20 min, and then the medium was changed to Biolog MAS to simulate reperfusion in the absence or presence of 50 μM PK11195. MAS contained a redox dye and low concentration of digitonin required for plasma membrane permeabilization in order to allow pre-loaded cytosolic and mitochondrial substrates and intermediates to enter the cells. Next, cardiomyocytes were loaded into the 96-well Biolog MitoPlates to examine the major changes in glycolytic metabolites (Figure 5), mitochondrial Krebs (TCA) cycle substrates (Figure 6), amino/fatty acids, and ketone intermediates (Figure 7) utilization.

Figure 5A shows a schematic representation of the main steps of glycolysis, pentose phosphate pathway, glycogenolysis, and gluconeogenesis pathways with the intermediates which were evaluated in our assay. As shown in Figure 5B, there was a significant decrease in utilization of glucose and glucose-6 phosphate (the product of the first reaction in glycolysis) upon reperfusion (see red bars) following cell exposure to 20 min of simulated ischemia compared to control (untreated conditions; black bars). In concert with decreased glucose utilization, pyruvate (the product of the last reaction in glycolysis) utilization was decreased in simulated post-ischemic reperfusion (red) compared to the control untreated cardiomyocytes (black). However, a significant increase in utilization of lactate was noted following simulated ischemia (red). In contrast, treatment with 50 μM PK11195 only during simulated reperfusion improved glucose, glucose-6 phosphate, pyruvate utilization, and decreased lactate utilization (blue).

However, the most significant change during I/R was an increase in the utilization of glycogen and glucose-1-phosphate, which is the product of glycogenolysis, indicating increased reliance on endogenous glucose sources (e.g., glycogen) in simulated ischemia and at the beginning of reperfusion. There was also increased utilization of D-gluconate-6-phosphate (an intermediate in pentose phosphate pathway) and D,L-α-glycerol-phosphate (an intermediate of the glycerol-3-phosphate shuttle which translocates electrons generated during glycolysis across the inner mitochondrial membrane for oxidative phosphorylation). In contrast, treatment with 50 μM PK11195 on reperfusion reversed the changes observed with simulated I/R, suggesting decreased reliance of the heart on the internal energy sources such as glycogen in order to sustain anaerobic glycolysis.

Next, using TCA cycle metabolites pre-loaded into the assay plate, we determined that there was a significant decrease in utilization of early TCA cycle intermediates such as citrate, cis-aconitate, isocitrate, and α-ketoglutarate with I/R (Figures 6A,B). Despite that, there was a significant increase in succinate oxidation (247% of untreated cardiomyocytes) following I/R (Figure 6B). This increase in succinate utilization was significantly prevented by cell treatment with 50 μM PK11195 applied during the reperfusion phase (97% of control untreated cells). Of note, the utilization of fumarate (−68%) and malate (−53%) was significantly decreased in cardiomyocytes exposed to I/R, and increased in PK11195 samples treated during simulated reperfusion. This suggests that the observed increase in succinate utilization could be due to increased succinate formation *via* anaplerosis.

The Biolog assay also allowed us to examine utilization of several amino acids. We found reduced utilization of L-glutamine (−43%) and L-glutamate (−60%), which are precursors for the TCA cycle anaplerotic entry point at the level of α-ketoglutarate, following 20 min of simulated ischemia compared to control cells. Cardiomyocytes treatment with PK11195 during simulated reperfusion increased utilization of both L-glutamine (+54%) and L-glutamate (+32%) even above the levels observed in control cells (Figure 7). Since α-ketoglutarate and amino acids entering the TCA cycle at the level of α-ketoglutarate were significantly decreased following 20 min simulated ischemia, it is reasonable to hypothesize that the amino acids (isoleucine, methionine, and threonine) and fatty acids intermediates that enter the TCA cycle anaplerotic entry point at the level of succinyl-CoA (Figure 6A) might contribute to succinate formation during ischemia and subsequent utilization during reperfusion. Furthermore, it is known that elevated levels of branched chain amino acids (BCAAs), such as valine, leucine, and isoleucine, contribute to IRI (Wang et al., 2016; Li et al., 2017). With the Biolog assay, we were able to monitor leucine utilization. As shown in Figure 7, the utilization of leucine was significantly increased in cardiomyocytes following 20 min of simulated ischemia, suggesting accumulation of leucine during ischemia. We also determined that utilization of α-ketoisocaproic acid (KIC), a metabolite of leucine, was decreased following 20 min of simulated ischemia. This indicates that either conversion of leucine to α-ketoisocaproic acid was decreased during simulated ischemia or α-ketoisocaproic acid was converted to leucine during ischemia due to enhanced activity of branched-chain aminotransferase 2 enzyme (BCAT2), an enzyme that catalyzes the reversible transamination of α-ketoisocaproic acid back to leucine (Moghei et al., 2016). Cardiomyocytes incubated with PK11195 during simulated reperfusion showed decreased utilization of leucine and reversed utilization of α-ketoisocaproic acid to the pre-ischemic levels. Increased utilization of leucine was reported to inhibit insulin-stimulated glucose transport (Moghei et al., 2016). Furthermore, experiments performed in mice with genetic BCAAs catabolic defects demonstrated that chronic accumulation of BCAAs increased fatty acid oxidation, enhanced lipid peroxidation, and sensitized hearts to IRI (Li et al., 2020). We, therefore, examined utilization of palmitoyl-D,L-carnitine and found that its utilization was indeed significantly increased following 20 min of simulated ischemia and normalized by addition of PK11195 on reperfusion (Figure 7). Although the γ-amino-butyric acid (GABA) shunt potentially can contribute to succinate formation, we did not observe any major changes in GABA utilization (Figure 7). We also did not see significant changes in D-L-β-hydroxybutyrate and α-keto-butyric acid utilization (Figure 7). α-Ketobutyrate is one of the degradation products of the amino acid threonine (see Figure 6A), which can also be generated by homocysteine degradation and the methionine metabolism. Upon transport into the mitochondrial matrix, α-ketobutyrate is converted to propionyl-CoA by branched-chain alpha-keto acid dehydrogenase complex (BCKDC), and then to succinyl-CoA. BCKDC is a member of the mitochondrial α-ketoacid dehydrogenase complex family which consists of pyruvate dehydrogenase and α-ketoglutarate dehydrogenase. Utilization of the ketone body D-L-β-hydroxybutyrate has been reported to be suppressed in cardiac ischemia (Arima et al., 2019); however, we did not find major changes in D-L-β-hydroxybutyrate utilization during reperfusion either in the presence or absence of PK11195.

To summarize, our cellular model of simulated ischemia and reperfusion revealed changes in cardiac glucose, amino acids, and fatty acid metabolism similar to that reported in human (Fillmore et al., 2014; Yehualashet et al., 2020) and animal models (Masoud et al., 2014; Heitzman et al., 2020) of ischemia and reperfusion. We found a significant increase in succinate and glycogen utilization during reperfusion which was reversed by PK11195. Furthermore, a significant decrease in α-ketoglutarate utilization was noted, together with the corresponding decrease in glutamine and glutamate metabolism which contribute to α-ketoglutarate formation (see Figure 7A). PK11195 also restored impaired glutamine-α-ketoglutarate utilization. Overall, PK111951 administration during reperfusion resulted in a shift in metabolism which led to the restoration of glucose consumption and a decrease in fatty acid oxidation.

### Succinate-Induced ROS Generation Was Significantly Decreased by PK11195 Treatment

**Figure 8:**
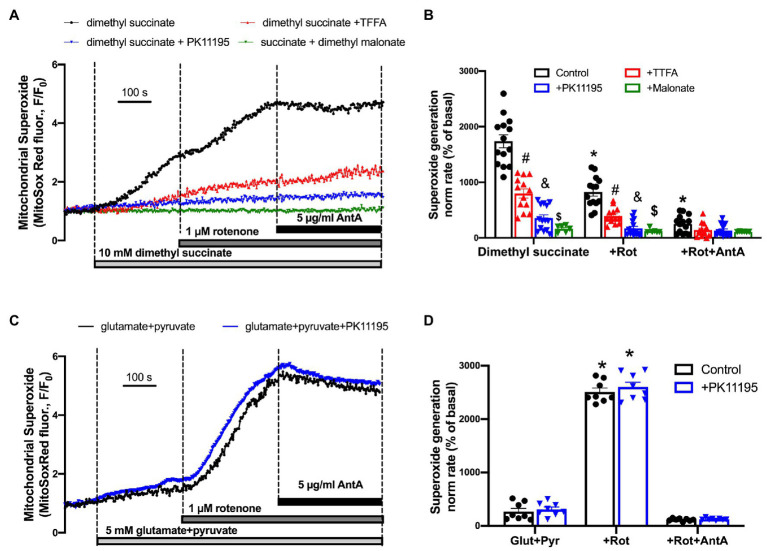
Succinate-mediated ROS generation in control ventricular rabbit cardiomyocytes. **(A)** Shown are representative traces of mitochondrial superoxide generation measured from control rabbit myocytes exposed to 10 mM cell-permeable dimethyl succinate (black trace) and myocytes treated with 100 μM TTFA (red trace), 50 μM PK11195 (blue trace), and 10 mM dimethyl malonate (green trace). About 1 μM rotenone and 5 μg/μl antimycin A (AntA) were added sequentially in each experiment to block mitochondrial complex I and complex III, respectively. **(B)** Summary bars reflect that superoxide generation induced by dimethyl succinate was only partially affected by complex I inhibitor rotenone, and almost completely blocked by complex III inhibitor AntA indicating the forward mode of superoxide production. PK11195 was more effective in blocking mitochondrial superoxide generation compared to complex II inhibitor, TTFA but less effective than dimethyl malonate. Data expressed as mean ± SEM. *n* = number of cells from three different animals per each group. **(C)** Changes in mitochondrial superoxide production upon complex I stimulation with 5 mM glutamate in combination with 5 M pyruvate, and subsequent exposure to 1 μM rotenone and 5 μg/μl AntA. **(D)** Summary of superoxide changes upon complex I stimulation. ^*^p < 0.05 reflects a significance in substrate treated cells (dimethyl succinate or glutamate/pyruvate) in the absence and presence of 1 μM Rot and 1 μM Rot + 5 μg/μl Ant A; ^#^*p* < 0.05 reflects a significance between TTFA treated vs. untreated cells (dimethyl succinate and dimethyl succinate + Rot only); ^&^*p* < 0.05 reflects a significance between PK11195 treated cells vs. untreated groups (dimethyl succinate and dimethyl succinate + Rot only); ^$^*p* < 0.05 reflects a significance between malonate treated cells vs untreated groups (dimethyl succinate and dimethyl succinate + Rot only).

In order to test whether succinate utilization during reperfusion was the main source of ROS, we performed experiments where control rabbit myocytes were exposed to a high concentration of cell-permeable succinate, 10 mM of dimethyl succinate. Cells were loaded with superoxide-sensitive fluorescent indicator MitoSox Red to monitor superoxide generation in mitochondria. All cells were electrically-field stimulated at 0.5 Hz to maintain excitation-contraction coupling. Figure 8A shows a representative trace of mitochondrial superoxide generation measured from control rabbit myocytes exposed to dimethyl succinate, and then sequentially exposed to 1 μM rotenone and 5 μg/μl antimycin A (AntA) to block mitochondrial complex I and complex III, respectively (black trace). As evident in Figure 8B, the rate of superoxide generation was only partially blocked by the complex I inhibitor rotenone, indicating that a portion of the superoxide originated from reverse electron flow from the mitochondrial complex II to mitochondrial complex I. However, the rate of increase was almost completely blocked by complex III inhibition with AntA, indicating a forward mode of superoxide production (Quinlan et al., 2012). In a separate set of experiments, cells were preincubated with 100 μM TTFA (red trace), which inhibits mitochondrial complex II by binding to two ubiquinone binding sites, Qp and Qd (Sun et al., 2005), 50 μM PK11195 (blue trace), and 10 mM dimethyl malonate, a competitive inhibitor of the mitochondrial complex II (green trace) which binds at the succinate binding site (Drose, 2013). Mitochondrial complex II inhibition with 100 μM TTFA resulted in significant inhibition of mitochondrial superoxide generation, confirming that the majority of superoxide generation was due to succinate oxidation at the level of mitochondrial complex II. However, when cells were treated with 50 μM PK11195, the succinate-induced increase in superoxide generation was almost completely abolished. When we used a competitive inhibitor of complex II, dimethyl malonate, we observed a maximal inhibitory effect of the mitochondrial superoxide generation. These data agree with data presented in Figure 4A where 50 μM PK11195 effectively blocked ROS production when applied during the reperfusion. Based on the data presented in Figure 6, we conclude that PK11195 inhibits succinate-induced superoxide generation *via* inhibition of succinate utilization. The exact mechanism will require further investigation.

Interestingly, we did not observe any significant effect of PK11195 on superoxide generation when mitochondrial complex I activity was stimulated by 5 mM glutamate in combination with 5 mM pyruvate. The rate of mitochondrial superoxide generation was much lower (Figure 8D) compared to that found with succinate (Figure 8B; *p* < 0.05). However, when mitochondrial complex I was blocked with 1 μM Rotenone, the maximal superoxide generation was observed in ventricular myocytes, which was blocked by inhibiting complex III of the mitochondrial respiratory chain with AntA. These data suggest that PK11195 did not act as a superoxide scavenger, and its effects were specific for succinate availability and utilization at mitochondrial complex II.

## Discussion

This study has elucidated key actions of PK11195, which is reported to bind to TSPO and change its conformation and activity (Le Fur et al., 1983a,b; Mestre et al., 1985; Rosenberg et al., 2011; Jaremko et al., 2014; Azarashvili et al., 2015; Yasin et al., 2017), on mitochondrial function and cell survival during simulated I/R in rabbit cardiomyocytes. We demonstrated that administration of PK11195 did not provide any protective effect when given during both ischemia and reperfusion; however, PK11195 applied only during reperfusion decreased cell death, prevented ROS-induced ROS generation and the resultant loss of the mitochondrial membrane potential. This protection was characterized by maintenance of [Ca^2+^]_m_ during reperfusion coupled with limited succinate oxidation. Together, these data suggest a protective role of PK11195 during reperfusion following an ischemic episode.

Transporter protein expression responds to injury, increasing almost 3-fold in experimental heart failure in mice (Thai et al., 2018). This increase was associated with decreased mitochondrial Ca^2+^ uptake and bioenergetics. Our earlier study demonstrated that the endogenous TSPO ligand hemin (protoporphyrin IX) dose-dependently (2.5–10 μM) reduced Ca^2+^ uptake in isolated heart mitochondria, possibly by modulating the open probability of the VDAC (Tamse et al., 2008). When the complex of VDAC, TSPO, and the inner membrane ANT was isolated from the rat heart mitochondria and incorporated in the lipid bilayer, hemin was able to change the observed channel activity from open to closed in dose-dependent manner (Tamse et al., 2008). At concentrations above 2.5 μM, a dual effect of hemin was observed: the initial decrease in mitochondrial Ca^2+^ uptake was accompanied by the increased CsA-sensitive mitochondrial Ca^2+^ release. Classical studies from Marco Colombini’s group (Tan and Colombini, 2007) demonstrated that Ca^2+^ passes through the outer mitochondrial membrane *via* VDAC with higher permeability (up to 10 times) observed in the closed states (i.e., states with lower permeability to metabolites) than that in the open state. In agreement with that, (Azarashvili et al., 2015) showed that a combination of VDAC channel closure with 5 μM G3139 and TSPO inhibition with 100 nM PK11195 led to the highest permeability of the VDAC for Ca^2+^ in rat brain mitochondria, eventually leading to the opening of the mPTP. Interestingly, addition of 100 nM PK11195 to mitochondria alone effectively suppressed mPTP opening. Similar effects were observed when anti-TSPO antibody was used to block TSPO. However, this study also found that using a higher concentration of PK11195 (50 μM, a dose which is also used in our study) induced a maximal mitochondrial Ca^2+^ uptake and mPTP activation which was not affected by the VDAC inhibitor G3139. These observations led to the conclusion that VDAC could also be a potential target of PK11195 when used at a higher concentration. However, it could be also explained by the fact that 50 μM PK11195 induced the highest mitochondrial Ca^2+^ uptake in brain mitochondria. The Ca^2+^ sensitivity of cardiac mitochondria for mPTP activation could be different compared to brain mitochondria.

The involvement of TSPO in mPTP formation was ruled out in the studies using TSPO KO animals. We (Thai et al., 2018) and others (Sileikyte et al., 2014) did not find any difference in Ca^2+^-induced mPTP opening in permeabilized cardiomyocytes or calcium retention capacity in isolated cardiac mitochondria from cardiospecific TSPO KO and corresponding WT control mice. This, however, cannot exclude that TSPO and its modulators could affect mPTP activity indirectly. For example, several studies demonstrated that TSPO ligands 4'-chlorodiazepam and TRO40403 delayed mPTP opening following I/R due to decrease in mitochondrial cholesterol accumulation (Paradis et al., 2013; Musman et al., 2017) and associated decrease in the formation of auto-oxidized oxysterols (Paradis et al., 2013). Auto-oxidized oxysterols are formed due to cholesterol oxidation by ROS (Paradis et al., 2013) and affect membrane fluidity and permeability (Targosz-Korecka et al., 2020). Furthermore, a direct TSPO-independent inhibitory effect of PK11195 on F_0_F_1_-ATP synthase at oligomycin sensitivity conferral protein (OSCP) subunit (Cleary et al., 2007) has been demonstrated. Currently, F_0_F_1_-ATP synthase (Bonora et al., 2013; Alavian et al., 2014; Mnatsakanyan et al., 2019) is proposed as one of the main candidates for mPTP formation in addition to the inner membrane ANT (Brustovetsky and Klingenberg, 1996; Karch et al., 2019). Several subunits of F_0_F_1_-ATP synthase were proposed as possible candidates for the mPTP channel (Giorgio et al., 2013, 2017; Alavian et al., 2014; He et al., 2017a,b); however, controversies still exists (He et al., 2017a,b; Bernardi, 2020) about the exact mechanism and subunits involved in mPTP formation. The well-established regulatory protein of the mPTP, cyclophilin D (Javadov and Kuznetsov, 2013), which favors mPTP opening, interacts with both ANT and the OSCP subunit of F_0_F_1_-ATP synthase. Cyclophilin D is inhibited by CsA, and the fact that PK11195 can interact and inhibit the OSCP subunit of F_0_F_1_-ATP synthase suggests a possible direct effect of PK11195 on the mPTP.

As shown in Figure 3, application of 50 μM PK11195 during reperfusion prevented large-scale oscillations in the mitochondrial membrane potential during 3 h of observation time. This effect was much stronger compared to the effect observed with CsA treatment, a known mPTP desensitizer. In contrast to the general assumption that mitochondrial Ca^2+^ overload during reperfusion triggers mPTP opening (Halestrap, 2006; Hurst et al., 2017), we found a significant decrease in mitochondrial Ca^2+^ concentration in cardiac mitochondria during simulated reperfusion in control (drug-untreated) myocytes (see Figures 2, 4B). Furthermore, other studies using ratiometric dyes Fura-2 and Indo-1 (Abdallah et al., 2010; Andrienko et al., 2016) also demonstrated a sustained increase in [Ca^2+^]_m_ during ischemia and then an abrupt increase in [Ca^2+^]_m_ during reperfusion followed by a decline in [Ca^2+^]_m_. Therefore, we believe that the increase in mitochondrial Ca^2+^ uptake observed with PK11195 treatment on reperfusion contributed to the maintenance of mitochondrial bioenergetics rather than inducing mPTP on reperfusion. In contrast, application of 50 μM PK11195 during both ischemia and reperfusion, led to a massive increase in mitochondrial Ca^2+^ during ischemia with a subsequent decline in [Ca^2+^]_m_ during reperfusion time (see Figure 2). The exact mechanism of Ca^2+^ uptake modulation by TSPO action is unclear. However, it was suggested by Campanella’s group that the inhibition of mitochondrial Ca^2+^ uptake by TSPO is a consequence of the phosphorylation of the voltage-dependent anion channel 1 (VDAC1) by the protein kinase A (PKA), which is recruited to the mitochondria by TSPO in complex with the Golgi Acyl-CoA binding domain containing 3 (ACBD3) protein (Gatliff et al., 2017).

Prior studies of cardiac I/R using PK11195 are limited. Mestre et al. (1985) administered PK11195 to dogs in doses from 5 to 25 mg/kg *via* intradermal injections and showed protection against both early and delayed ventricular arrhythmias induced by 20 min of ischemia and against ventricular fibrillation following reperfusion. Our current data demonstrate that the effect of PK11195 was different depending on whether the drug was added during both ischemia and reperfusion or only during reperfusion. Addition of PK11195 during both ischemia and reperfusion did not protect cardiomyocytes from cell death following I/R and even exacerbated cell death during ischemia (Figure 1), coupled with significantly increased mitochondrial Ca^2+^ uptake (Figures 2, 4) and ROS generation (Figure 4) with subsequent loss of mitochondrial Ca^2+^ during reperfusion phase (Figure 2). However, when PK11195 was added at the onset of reperfusion and present during reperfusion phase, mitochondrial Ca^2+^ levels were preserved at pre-ischemic levels (Figure 2). Moreover, PK11195 was even more effective in preventing large-scale ΔΨ_m_ oscillations compared to the mPTP desensitizer, cyclosporin A (Figure 3). These oscillations typically were observed after ~1 h of reperfusion and led to cell death if untreated. These large-scale oscillations were originally described by Zorov et al. (2000) and attributed to RIRR. Excessive exposure to oxidative stress and a decrease in ROS-scavenging enzyme activities eventually led to ROS accumulation at the threshold levels that can trigger opening of large mitochondrial channels (IMAC or mPTP), simultaneous collapse in ΔΨ_m_, and ROS release in cytosol (Zorov et al., 2006). This triggers a chain of events leading to RIRR propagation *via* cardiomyocyte which was associated with large-scale oscillations in mitochondria involved in post ischemic arrhythmias and cellular injury (Akar et al., 2005). Prior studies (Robin et al., 2007; Loor et al., 2011) have also demonstrated that ROS generation during ischemia primes cardiomyocytes for cell death during reperfusion. It was determined that ischemia significantly depleted reduced to oxidized glutathione ratio (GSH/GSSG) levels with further GSH/GSSG decline noted during reperfusion (Robin et al., 2007; Loor et al., 2011; Seidlmayer et al., 2015). Our data presented in Figures 1–4 also support this hypothesis.

To explore potential mechanisms of the protective effect of PK11195, we measured energetic pathways using the Biolog system. We found that PK11195 significantly decreased succinate utilization following I/R (Figure 6) and succinate-induced ROS generation (Figure 8). Since excessive succinate accumulation during ischemia was reported to drive reperfusion injury through mitochondrial ROS generation (Chouchani et al., 2014), inhibition of succinate accumulation is a potential mechanism to explain the beneficial effect of PK11195. However, the precise manner in which TSPO affects succinate utilization is not known. One potential mechanism implies TSPO modulation of VDAC which is involved in Ca^2+^, nucleotide (ATP/ADP and NADH/NAD^+^), and metabolite (glutamate, citrate, pyruvate, malate, and succinate) transport into and out of mitochondria (Shoshan-Barmatz et al., 2019). It was previously demonstrated (Kurz et al., 2015) that ΔΨ_m_ oscillations depend on the presence of metabolic substrates, with a higher frequency of oscillations observed in cardiomyocytes and intact hearts perfused with glucose and pyruvate compared to that observed with lactate and β-hydroxybutyrate. It is known that overexpression of TSPO results in inhibition of VDAC1 function, and pharmacological or genetic inhibition of TSPO enhances VDAC1 activity (Gatliff et al., 2014). Furthermore, TSPO knockout increases mitochondrial Ca^2+^ uptake in cardiac myocytes from the failing heart (Thai et al., 2018), and, therefore, can enhance energy production *via* activation of Ca^2+^-dependent pyruvate dehydrogenase, isocitrate dehydrogenase, and α-ketoglutarate dehydrogenase in the TCA cycle.

A prior study postulated that ischemic succinate accumulation originates from the reversal of succinate dehydrogenase, which in turn is driven by fumarate overflow from purine nucleotide breakdown and partial reversal of the malate/aspartate shuttle (Chouchani et al., 2014). During reperfusion, the accumulated succinate is rapidly re-oxidized by succinate dehydrogenase, driving extensive ROS generation by reverse electron transport at mitochondrial complex I. The follow-up study from Paul Brookes group, however, proposed a bifunctional role of succinate during I/R (Zhang et al., 2018). It was suggested that during ischemia, the accumulation of succinate was mainly complex II-independent, originating mainly from canonical Krebs cycle activity, partly supported by aminotransferase anaplerosis and glycolysis from glycogen. Enhancing canonical Krebs cycle activity with cell-membrane permeable dimethyl-α-ketoglutarate enhanced succinate accumulation and improved cardiac bioenergetics during ischemia. In contrast, complex II-dependent oxidation at reperfusion was associated with increase in detrimental events (increased oxidative stress and increase in the infarct size; Zhang et al., 2018). Inhibition of mitochondrial complex II with atpenin A5 at the onset of reperfusion decreased augmented succinate oxidation and prevented IRI in the heart (Zhang et al., 2018).

In the current experiments, succinate-induced ROS generation was only partially affected by complex I inhibition with rotenone (−40%) and almost completely abolished by the complex III inhibitor AntA (−60%; Figure 8). This indicates that even though reverse mode of electron flow (RET) contributed to succinate-induced ROS generation, the majority of ROS was generated due to elevated electron flow *via* respiratory chain operating in forward mode (FET).

We also demonstrated that PK11195 diminished a significant reliance on glycogen utilization, which possibly contributed to succinate formation during ischemia as suggested by Zhang et al. (2018). Our earlier study (Schaefer and Ramasamy, 1997) demonstrated that increased glycogen utilization *via* glycogenolysis was required to maintain cardiac energetics during ischemia and was associated with better functional recovery on reperfusion, but only in the hearts from fasted rats. Interestingly, the increased glycogen levels in fed rats perfused with insulin to increase glucose uptake resulted in worse functional recovery compared control untreated rats.

In our study, we observed a significant increase in glycogen and D-L-α-glycerol-PO_4_ (Figure 5) utilization during reperfusion which was reversed by PK11195 treatment. Interestingly, D-L-α-glycerol-PO_4_ is the part of the glycerol/FFA cycle which intersects between glucose, TCA, and fatty acids metabolism (see schematic in Figure 5A; Mugabo et al., 2016; Possik et al., 2017). As shown in Figure 7A, TSPO could be a part of the multi-protein fatty acid transfer complex on the outer mitochondrial membrane consisting of TSPO, VDAC, long chain acyl-CoA synthetase (ACSL), and carnitine palmitoyltransferase 1 (CPT1). It was shown that TSPO could be isolated in complex with VDAC and ANT (Gatliff et al., 2014; Shoshan-Barmatz et al., 2019). Furthermore, the evidence for VDAC/ACSL/CPT1 complex was presented by Charles Hoppel’s group (Lee et al., 2011). It is plausible to speculate that TSPO can participate in fatty acid transfer at the outer mitochondrial membrane and its inhibition can shift metabolism toward glucose utilization. Furthermore, as discussed before, modulation of VDAC permeability by TSPO can affect succinate and amino acids delivery.

Interestingly, the effect of PK11195 on succinate-induced ROS generation was stronger than the complex II inhibitor TFFA, but comparable to the effect of the complex II inhibitor malonate. TTFA binds to the Qp site of the mitochondrial complex II located between subunit B and subunits C and D (Drose, 2013). In contrast, malonate is a competitive inhibitor of the mitochondrial complex II which binds to the carboxylate (succinate) binding site of subunit A (Drose, 2013). Therefore, PK11195 may act by decreasing substrate (i.e., succinate) availability for mitochondrial complex II. It is known that complex II activity depends on succinate concentration with K_m_ values ~1 mM (Ackrell et al., 1978). However, it has been reported that during ischemia, succinate concentration can increase 6–12 times relative to normoxia (Chouchani et al., 2014; Zhang et al., 2018). This increase in the succinate pool during ischemia could occur due to (1) increased supply of glutamate *via* α-ketoglutarate by transamination (Zhang et al., 2018); (2) partial reversal of mitochondrial TCA cycle during ischemia and complex II working as fumarate reductase (Hohl et al., 1987; Ralph et al., 2011; Chouchani et al., 2014); or (3) potentially by increased delivery of succinate to mitochondria *via* VDAC.

## Limitations

While clearly demonstrating a protective role of PK11195 applied during reperfusion, it is recognized that this study used a model of simulated I/R in quiescent cardiomyocytes and, thus with low energy demands. Therefore, further validation in *in vivo* and large animal studies is required to account for the extent and substrate utilization changes in the working heart. A single concentration of PK11195 was employed based on prior studies (Stadulyte et al., 2019) and thus it is unknown whether different concentrations would have greater or lesser effects. Several reports indicate that PK11195 can affect lipid fluidity of mitochondrial outer membrane, and, therefore, can potentially affect the function of other proteins in OMM (Miccoli et al., 1999; Hatty et al., 2014) or even directly inhibit the OSCP subunit of F_0_F_1_-ATP synthase (Cleary et al., 2007; Krestinina et al., 2009) which is reported to form mPTP (Alavian et al., 2014; Mnatsakanyan et al., 2019; Bernardi, 2020). Finally, while inhibition of increased succinate oxidation on reperfusion provides an explanation for the beneficial effect of PK11195, the exact mechanism by which PK11195 limits succinate oxidation is unknown and requires further investigation.

## Conclusion

In summary, using a model of simulated I/R, we demonstrated that PK11195 administration only at reperfusion limited cell death, prevented loss of [Ca^2+^]_m_ and flickering in mitochondrial membrane potential due to ROS-induced ROS generation. Measurement of bioenergetics showed that PK11195 also normalized succinate oxidation and glutamate utilization when applied at the onset of reperfusion. In contrast, when PK11195 was also present during ischemia (as well as reperfusion), it did not provide any protection. These data emphasize the differences in PK11195 effects in terms of cell survival during I/R with careful attention to the timing of administration – the protective effect was observed only when the drug was administered at the onset and during reperfusion.

## Data Availability Statement

The raw data supporting the conclusions of this article will be made available by the authors, without undue reservation.

## Ethics Statement

All animal handling and laboratory procedures were performed in accordance with the approved protocols of the Institutional Animal Care and Use Committee of the University of California, Davis conforming to the Guide for the Care and Use of Laboratory Animals published by the United States National Institute of Health (8th Edition, 2011).

## Author Contributions

ED designed and supervised this study and wrote the manuscript. LS, BH, PT, and ED performed the experiments and analyzed the data. SS and DB edited the manuscript and provided the financial support. There are no competing financial interests. All authors contributed to the article and approved the submitted version.

### Conflict of Interest

The authors declare that the research was conducted in the absence of any commercial or financial relationships that could be construed as a potential conflict of interest.

## Abbreviations

ACSL: Long chain acyl-CoA synthetase
ANT: Adenine nucleotide transporter
AntA: Antimycin A
[Ca^2+^]_i_: Cytosolic calcium concentration
[Ca^2+^]_em_: Extramitochondrial free calcium
ETC: Electron transport chain
FCCP: Carbonyl cyanide 4-(trifluoromethyhoxy)-phenylhydrazone
HF: Heart failure
I/R: Ischemia/reperfusion
IRI: Ischemia/reperfusion injury
MI: Myocardial infarction
MCU: Mitochondrial Ca^2+^ uniporter
mPTP: Mitochondrial permeability transition pore
PK11195: Isoquinoline carboxamide
ROS: Reactive oxygen species
TSPO: Transporter protein of the outer mitochondrial membrane
TTFA: Thenoyltrifluoroacetone
ΔΨ_m_: Mitochondrial membrane potential
VDAC: Voltage-dependent anion channel
